# Phase Conjugated and Transparent Wavelength Conversions of Nyquist 16-QAM Signals Employing a Single-Layer Graphene Coated Fiber Device

**DOI:** 10.1038/srep22379

**Published:** 2016-03-02

**Authors:** Xiao Hu, Mengqi Zeng, Yun Long, Jun Liu, Yixiao Zhu, Kaiheng Zou, Fan Zhang, Lei Fu, Jian Wang

**Affiliations:** 1 Wuhan National Laboratory for Optoelectronics, School of Optical and Electronic Information, Huazhong University of Science and Technology, Wuhan 430074, Hubei, China; 2 College of Chemistry and Molecular Science, Wuhan University, Wuhan 430074, Hubei, China; 3 State Key Laboratory of Advanced Optical Communication Systems and Networks, Peking University, Beijing 100871, China

## Abstract

We fabricate a nonlinear optical device based on a fiber pigtail cross-section coated with a single-layer graphene grown by chemical vapor deposition (CVD) method. Using the fabricated graphene-assisted nonlinear optical device and employing Nyquist 16-ary quadrature amplitude modulation (16-QAM) signal, we experimentally demonstrate phase conjugated wavelength conversion by degenerate four-wave mixing (FWM) and transparent wavelength conversion by non-degenerate FWM in graphene. We study the conversion efficiency as functions of the pump power and pump wavelength and evaluate the bit-error rate (BER) performance. We also compare the time-varying symbol sequence for graphene-assisted phase conjugated and transparent wavelength conversions of Nyquist 16-QAM signal.

Graphene^1^, a single layer of carbon atoms arranged in a hexagonal honeycomb lattice, is a basic building block of well-known carbon materials such as graphite, carbon nanotubes and fullerene. Graphene has attracted a high level of research interest because of its exceptional electronic and photonic properties^2^,^3^,^4^,^5^. It has linear, massless band structure E_±_(p) = ±V|p|, where the upper (lower) sign corresponds to the electron (hole) band, p is the quasi-momentum, and V ≈ 10^6^ m/s is the Fermi velocity. Recently, a variety of graphene-based photonic devices have been reported, including wide bandwidth polarizers^6^, ultrafast photodetectors^7^,^8^, broadband modulators^9^,^10^, highly sensitive sensors^11^, ultrafast and ultra-broadband pulsed lasers^12^,^13^,^14^,^15^,^16^. Due to possessing linear band structure that allows interband optical transitions at ultrabroadband range (across the infrared and visible range), graphene has been suggested as a material that might have large χ^(3)^ nonlinearities^17^. As the nonlinear response of graphene is essentially dispersionless over the wavelength and much stronger compared to bulk semiconductors, it is naturally adaptable for FWM process. FWM has been observed in graphene in various configurations, e.g. slow-light graphene-silicon photonic crystal waveguide^18^, graphene optically deposited onto fiber ferrules^19^, and graphene based on microfiber^20^,^21^. Very recently, Xu and co-workers^22^ have reported an experimental observation of FWM-based wavelength conversion of a 10 Gb/s non-return-to-zero (NRZ) signal with mechanically exfoliated graphene.

It is well known that the increase of spectral efficiency has become an effective way when scaling up the data rate^23^,^24^. Advanced optical modulation formats have become of great importance to enable high-capacity optical transport networks^25^ where wavelength conversion function is highly desired. Nyquist pulse shaping signals have been widely used in wavelength-division multiplexing (WDM) systems. Moreover, Nyquist WDM technology can transmit a number of different wavelength channels in a single fiber and exhibit higher spectrum efficiency in contrast with conventional WDM. In previous FWM based tunable wavelength conversion^26^, the advanced modulation format is quadrature phase-shift keying (QPSK) with 2 bits information encoded in 1 symbol. In this work, the 16-ary quadrature amplitude modulation (16-QAM) signal with 4 bits information encoded in 1 symbol is adopted, which possesses higher spectral efficiency^27^,^28^. So, the combination of Nyquist pulse shaping signal and 16-QAM may be an attractive way to further increase the spectral efficiency. In this scenario, a laudable goal would be to develop wavelength conversion of Nyquist pulse shaping signals by exploiting graphene-assisted nonlinear optical device.

In this paper, considering the combination of advanced optical modulation format signal (Nyquist pulse shaping signal) and the optical nonlinearity of a single-layer graphene coated fiber device, we show an experimental observation of degenerate/non-degenerate FWM based wavelength conversion of a 5 Gbaud Nyquist 16-QAM signal. We study the wavelength tuning properties and the conversion efficiency as functions of the pump wavelength and pump power. Moreover, we characterize the performance of Nyquist 16-QAM wavelength conversion by measuring the BER as a function of the received optical signal-to-noise ratio (OSNR). Time-varying symbol sequence for phase conjugated wavelength conversion by degenerate FWM and transparent wavelength conversion by non-degenerate FWM are also measured for comparison.

## Results

### Experimental setup

**Figure 1:**
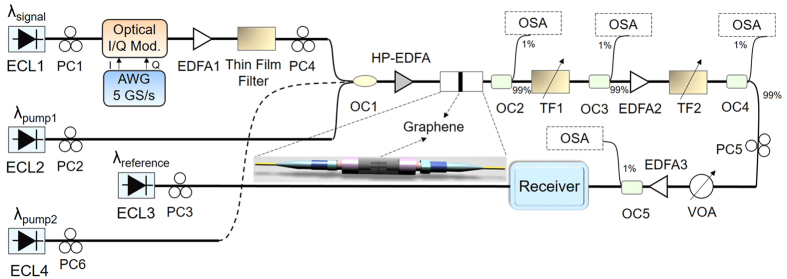
Experimental setup for wavelength conversion based on degenerate/non-degenerate FWM in graphene coated fiber device. Inset: “sandwiched structure” graphene sample used as a nonlinear optical device. ECL: external cavity laser; AWG: arbitrary waveform generator; TF: tunable filter; OC: optical coupler; PC: polarization controller; OSA: optical spectrum analyser.

Figure 1 shows the experimental setup for degenerate/non-degenerate FWM based wavelength conversion using a single-layer graphene coated fiber device. The continuous-wave (CW) output from an external cavity laser (ECL1) serves as the signal light for the degenerate/non-degenerate FWM process and is modulated with Nyquist 16-QAM signal at 5 Gbaud by a single-polarization optical in-phase/quadrature (I/Q) modulator. An arbitrary waveform generator (AWG) is used to produce the electrical signal. The modulated 5 Gbaud Nyquist 16-QAM signal is then amplified by an erbium-doped fiber amplifier (EDFA) followed by a thin film filter to suppress the amplified spontaneous emission (ASE) noise. Afterwards, the 5 Gbaud Nyquist 16-QAM signal is combined with a second CW pump from ECL2 for degenerate FWM and also a third CW pump from ECL4 for non-degenerate FWM through a coupler, amplified using a high-power EDFA (HP-EDFA), and launched into the single-layer graphene coated fiber device. The polarization states of the Nyquist 16-QAM signal and CW pumps are adjusted to achieve optimized conversion efficiency of degenerate/non-degenerate FWM in the single-layer graphene coated fiber device. The amplified Nyquist 16-QAM signal and CW pumps take part in the degenerate/non-degenerate FWM processes when passing through the single-layer graphene coated fiber device and newly converted idlers are generated, copying the data information carried by the signal. After the degenerate/non-degenerate FWM wavelength conversion process, the newly converted idler is selected using two tunable filters (TF1, TF2) for coherent detection. First, the newly converted idler is selected by use of TF1. The power level of the newly converted idler is relatively low, so the selected converted idler is amplified by EDFA2. Second, in order to suppress the ASE noise originated from EDFA2, another TF2 is used. That is, TF1 is used to select the newly converted idler, and TF2 is used to suppress the ASE noise. The CW output from ECL3 serves as a reference light for coherent detection. A variable optical attenuator (VOA) and a low-noise EDFA (EDFA3) are employed to adjust the received optical signal-to-noise ratio (OSNR) for BER measurements. An optical spectrum analyzer (OSA) is used to monitor the optical spectra.

**Figure 2:**
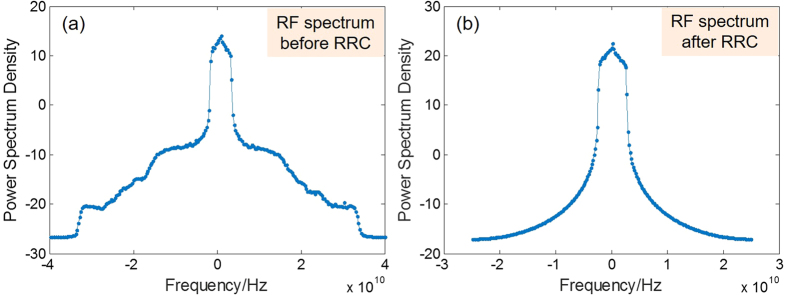
The RF spectrums of Nyquist pulse waveform (**a**) before RRC filter and (**b**) after RRC filter.

The line-width of ECL1 laser is 100 KHz. At the transmitter, the information data is firstly mapped into 16-QAM format and then packed into data frames. In each frame, 38400 data symbols are transmitted after the preamble. We insert 1 pilot in every 63 data symbols for phase recovery. The preamble includes two 63-symbol M-sequences as synchronization sequences and four 127-symbol M-sequences as training sequences. After 2 samples per symbol up-sampling, the signals are digitally shaped with a root-raised-cosine (RRC) filter. The roll-off factor of the RRC is 0.01. After digital to analog convertors (DAC), electrical low-pass filters with the bandwidth of 4.4 GHz are used as anti-aliasing filters to remove out-of-band radiation. At the receiver, a matched RRC filter is adopted. After synchronization, the signals are re-sampled to 2 samples per symbol and the training sequences are picked up for time-domain channel estimation and equalization. After equalization, the phase is corrected with pilots and further estimated with the blind phase search (BPS) algorithm. Finally, the bit error ratio (BER) is measured by error counting. The RF spectrums of Nyquist pulse waveform before RRC filter and after RRC filter is shown in Fig. 2.

### Experimental results

**Figure 3:**
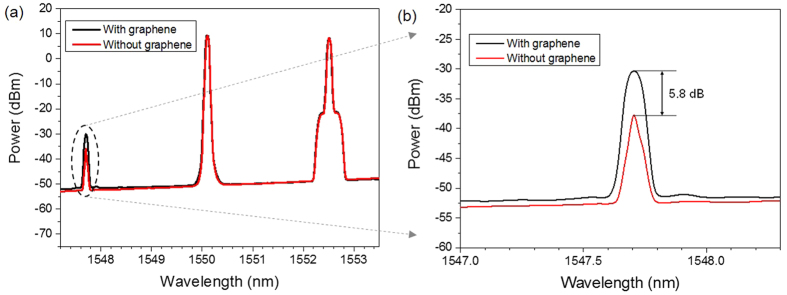
(**a**) Measured FWM spectra with (black) and without (red) graphene coated fiber device. (**b**) Inset: enlarged spectrum of converted idler.

We first demonstrate the wavelength conversion of Nyquist 16-QAM signal based on degenerate FWM process in the single-layer graphene coated fiber device. In the experiment, the signal wavelength is fixed at 1552.52 nm. Figure 3(a) shows a typical output degenerate FWM spectrum obtained after the single-layer graphene coated fiber device. A newly converted idler at 1547.71 nm is generated when the pump is tuned at 1550.12 nm. Here, we take degenerate FWM as an example and measure the output spectrum without graphene for reference under the same experimental conditions. Moreover, we repeat the experiment by adding extra 2 m and 5 m single mode fibers in the setup and get almost the same experimental results. As clearly shown in the Fig. 3(b) the power of converted idler without graphene is observed to be ~5.8 dB lower than the one with graphene. That is, under the same experimental conditions the converted idler without graphene is ~73.7% lower than the one with graphene. Therefore, the degenerate FWM in graphene contributes more in the wavelength conversion process.

**Figure 4:**
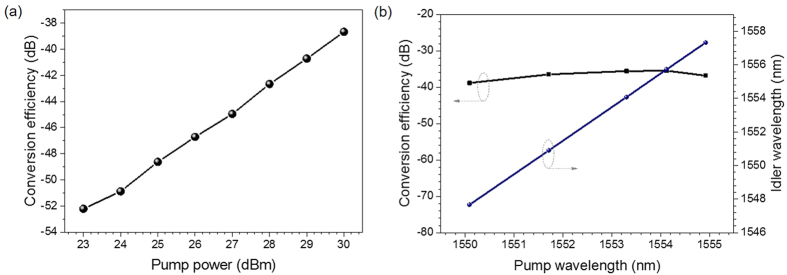
(**a**) Measured conversion efficiency versus pump power. (**b**) Measured conversion efficiency and converted idler wavelength versus pump wavelength.

We define the conversion efficiency as the power ratio of converted idler to signal. Figure 4(a) shows the measured degenerate FWM conversion efficiency as a function of the pump power (λ_pump_ = 1550.12 nm, λ_signal_ = 1552.52 nm). The conversion efficiency increases with the pump power. The conversion efficiency η of FWM can be approximately expressed as η = (**γ**∙Pp∙L)^2^, where **γ** is the effective nonlinear coefficient, Pp is the pump power, and L is the length. In fact, the conversion efficiency curve in Fig. 4(a) seems to follow a 2:1 ratio for the conversion efficiency versus pump at relatively high pump power above 25 dBm, while goes sub-quadratic at relatively lower pump power. Such interesting phenomenon might be ascribed to the saturable absorption effect of graphene. At lower pump power, the absorption by graphene limits the conversion efficiency, resulting in the sub-quadratic relationship between conversion efficiency and pump power. In contrast, at higher pump power, the absorption by graphene is saturable, and therefore the pump power dependent conversion efficiency follows a quadratic relationship which is in accordance with the theory of nonlinear coupled-mode equations under slowly varying envelope approximation and pump non-depletion approximation. Moreover, tunable operation of wavelength conversion is also studied. A CW pump at wavelength λ_pump_ and a data signal at wavelength λ_signal_ are combined together and sent into a graphene-assisted nonlinear device with high third order nonlinearity (χ^(3)^). Due to the energy conservation in the degenerate FWM process, the wavelength of the newly converted idler wave can be written by 1/λ_conv_ = 2/λ_pump_−1/λ_signal_. Hence, one can achieve tunable wavelength simply by adjusting the pump wavelength or the signal wavelength, i.e. variable λ_pump_ or λ_signal_ results in changeable λ_conv_. So the converted idler wavelength can be tuned simply by changing the pump wavelength, i.e. tunable wavelength conversion even for a fixed input signal wavelength. Figure 4(b) shows the measured degenerate FWM conversion efficiency of tunable wavelength conversion with graphene coated fiber device when the output power of HP-EDFA is fixed at 30.5 dBm. The signal wavelength is fixed at 1552.52 nm and the pump wavelength is tuned from 1550.09 nm to 1554.92 nm. A linear wavelength relationship between the converted idler and pump wavelength is observed. The conversion efficiency varies less than 3 dB within the pump wavelength tuning range.

**Figure 5:**
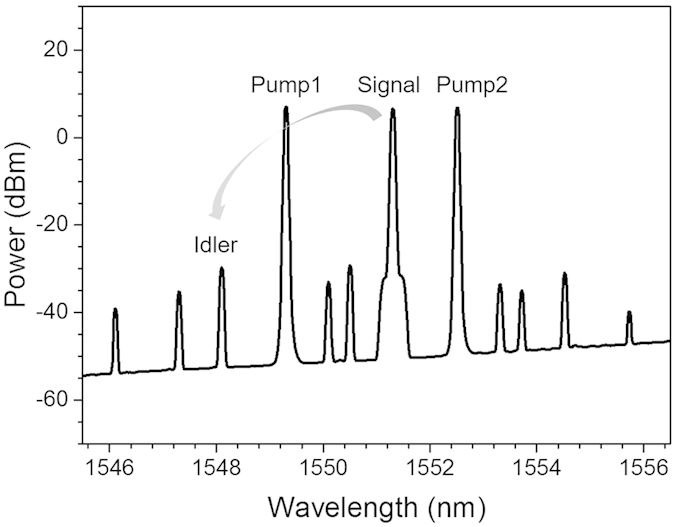
Measured non-degenerate FWM spectrum with graphene coated fiber device.

We then demonstrate the wavelength conversion of Nyquist 16-QAM signal based on non-degenerate FWM process in the single-layer graphene coated fiber device. Figure 5 shows a typical output non-degenerate FWM spectrum obtained after the single-layer graphene coated fiber device (λ_pump1_ = 1549.32, λ_pump2_ = 1552.52 nm, λ_signal_ = 1551.32 nm).

For the wavelength conversion based on degenerate FWM process as shown in Fig. 3, the electric field of the newly converted idler meets the relationship of E_idler_ ∝ E^2^_pump_E^*^_signal_, where E_idler_, E_pump_ and E_signal_ represent the complex electric fields of newly converted idler, input pump and input signal, respectively. “∗” denotes the complex conjugate of the electric field. Hence, the newly converted idler does not take the same data information carried by the original signal but its “phase conjugated” copy.

For the wavelength conversion based on non-degenerate FWM process as shown in Fig. 5, the electric field of the newly converted idler satisfies the relationship of E_idler_ ∝ E_pump1_E_signal_E^*^_pump2_, where E_pump1_ and E_pump2_ represent the complex electric fields of two input pumps. Therefore, the newly converted idler copies exactly the same data information carried by the original signal, i.e. fully transparent wavelength conversion.

**Figure 6:**
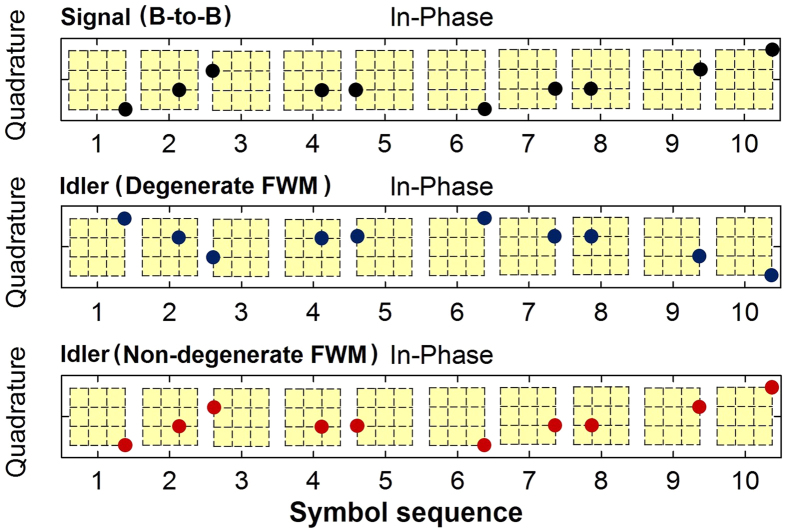
Time-varying symbol sequence of newly converted idlers by degenerate/non-degenerate FWM and original signal (B-to-B).

The converted idler is “phase conjugated” copy of the original data signal in the process of graphene-based degenerate FWM^26^. In this work, in order to verify the phase conjugated wavelength conversion by degenerate FWM and also transparent wavelength conversion by non-degenerate FWM, we measure, record and compare the typical time-varying symbol sequence of newly converted idlers by degenerate/non-degenerate FWM and original signal (back to back), as shown in Fig. 6. One can clearly see from Fig. 6 that the newly converted idler by degenerate FWM flips its constellation points in the complex I/Q plane with respect to the I-axis, corresponding to the phase conjugation of original Nyquist 16-QAM signal. In contrast, the newly converted idler by non-degenerate FWM duplicates the constellation of the original signal, corresponding to the transparent wavelength conversion of original Nyquist 16-QAM signal.

**Figure 7:**
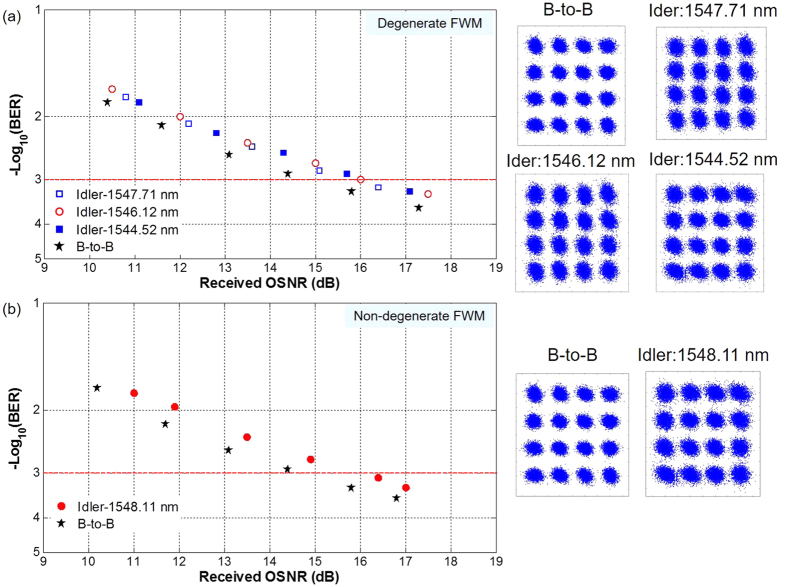
(**a**) Measured BER versus received OSNR for degenerate FWM wavelength conversion of Nyquist 16-QAM signal. (**b**) Measured BER versus received OSNR for non-degenerate FWM wavelength conversion of Nyquist 16-QAM signal. Insets show constellations of the B-to-B signals and newly converted idlers.

To characterize the performance of Nyquist 16-QAM wavelength conversion, we further measure the BER curve as a function of the received OSNR for back to back (B-to-B) signal and newly converted idler. Figure 7(a) plots measured BER performance for tunable Nyquist 16-QAM degenerate FWM wavelength conversion with the converted idler generated at 1547.71, 1546.12 and 1544.52nm, respectively. The power of HP-EDFA is set to be 31 dBm. The measured conversion efficiencies for newly converted idlers at 1547.71, 1546.12 and 1544.52 nm are −38.83, −42.47 and −50.21 dB, respectively. As shown in Fig. 7(a), the observed OSNR penalty is around 1 dB at a BER of 1 × 10^−3^ (7% forward error correction (FEC) threshold) for Nyquist 16-QAM wavelength conversion with the converted idler at 1547.71 nm. The received OSNR penalties of ~1.4 dB at a BER of 1 × 10^−3^ are observed for converted idlers at 1546.12 and 1544.52 nm. The increased OSNR penalty is mainly due to the reduced conversion efficiency for converted idlers at 1546.12 and 1544.52 nm. Figure 7(b) plots measured BER performance for tunable Nyquist 16-QAM non-degenerate FWM wavelength conversion with the newly converted idler generated at 1548.11 nm. The observed OSNR penalty is around 1.6 dB at a BER of 1 × 10^−3^. The right insets of Fig. 7 depict corresponding constellations of the B-to-B signals and newly converted idlers. The obtained results shown in Figs. 3, 4, 5, 6, 7 imply favorable performance achieved for wavelength conversion of Nyquist 16-QAM signal using a fiber pigtail cross-section coated with a single-layer graphene.

## Discussion

Luo and co-authors^29^ experimentally demonstrated that graphene can generate FWM. The graphene used in their experiment was prepared by the solution-based route. Then the graphene-polymer was transferred to the end face of optical fiber. However, in our work the single-layer graphene was grown by CVD method, which may possess higher quality than the solution-based route^30^. Actually, high-quality graphene also has high damage threshold. For the mechanically exfoliated graphene with high quality^22^, no significant damage is observed even applying an ultrahigh input power of 35 dBm (~3 W). In this work, the single-layer graphene is grown by CVD method. The fabricated and transferred graphene also shows high quality. In the experiment, we do not observe the damage of graphene (burned or punctured) even the input power is up to 33 dBm. So the damage threshold of the graphene coated optical fiber may ≥33 dBm. Additionally, the higher quality of graphene is of great benefit to larger χ^(3)^ nonlinearities. Therefore, the nonlinear optical device based on a fiber pigtail cross-section coated with a single-layer graphene grown by CVD might show favorable performance in practical applications.

Due to possessing linear band structure that allows interband optical transitions at ultrabroadband range (across the infrared and visible range), graphene has been suggested as a material that might have large χ^(3)^ nonlinearities^17^. As the nonlinear response of graphene is essentially dispersionless over the wavelength and much stronger compared to bulk semiconductors, it is naturally adaptable for FWM process. Moreover, we experimentally observe a maximum enhancement of 5.8 dB of conversion efficiency in the single-layer graphene coated fiber device, as shown Fig. 3(b). The enhancement mechanism can be explained as follows: For graphene-assisted nonlinear optical device, the total effective nonlinear Kerr coefficient is actually the combined contributions from the graphene and the device material (e.g. silicon in graphene-silicon waveguide, silica in graphene-coated fiber). The Kerr coefficients of silica in fiber, silicon and graphene are ~10^−20^ m^2^/W, ~10^−18^ m^2^/W and ~10^−11^ m^2^/W, respectively^31^,^32^,^33^. The third-order nonlinearity of graphene is several orders of magnitude larger than silica in fiber and silicon, which is due to the unique linear band structure of π-electrons^17^,^22^. Hence, the combined effective nonlinearity of graphene-assisted nonlinear optical device is increased and the graphene enhances the FWM process. Additionally, previous work has demonstrated that the nonlinear response is sensitive to the number of graphene layers^17^. So, it is possible to further enhance the degenerated and non-degenerated FWM response generated from different graphene layer by appropriately increasing the number of graphene layers employed in the experiment.

The phenomena of degenerate and non-degenerate FWM have been demonstrated in semiconductor optical amplifiers^34^ (SOAs), highly nonlinear fibers^35^ (HNLFs), silicon waveguide^36^. In the process of silicon waveguide based FWM wavelength conversion, two-photon absorption (TPA) and wavelength dispersion are important factors which have to be considered. The TPA induced free carrier absorption is strong at high pump powers. The nonlinear phase shift process of self-phase modulation (SPM) and cross phase modulation (XPM) may lead to the signal deterioration. The large third-order susceptibility χ^(3)^ of graphene could also give rise to nonlinear Kerr-effects such as FWM, TPA, stimulated Raman scattering (SRS), stimulated Brillouin scattering (SBS), SPM, and XPM. So, on may wonder whether the graphene based phase conjugated and transparent wavelength conversions still can occur, especially for advanced optical modulation formats signal, e.g., Nyquist 16-QAM. One may also wonder what is the influence of the XPM, SPM on the signal quality (e.g., signal-to-noise ratio degradation) in the process of graphene-assisted FWM based wavelength conversion of Nyquist 16-QAM signal. Previous experiments on enhanced FWM in graphene-assisted nonlinear optical devices have been demonstrated with impressive performance, such as the works by C. W. Wong and Y. J. Rao^18^,^20^,^21^,^37^,^38^. In this paper, we fabricate a graphene-coated fiber device with the graphene placed within the connector of two fibers, which is fully compatible with existing optical fiber transmission systems. We experimentally demonstrate phase conjugated wavelength conversion by degenerate FWM and transparent wavelength conversion by non-degenerate FWM in graphene. We also characterize the performance of Nyquist 16-QAM wavelength conversion by measuring the BER as a function of the received optical OSNR. The broadband nonlinear response, large χ^(3)^ nonlinearities, and compatibility with existing optical fiber transmission systems might enable novel architectures for optical signal processing applications.

## Conclusion

Firstly, FWM in graphene which is mechanically transferred on the end face of optical fiber has been experimentally observed. The degenerate/non-degenerate FWM based wavelength conversion of advanced modulation format signal (e.g. Nyquist 16-QAM) is further demonstrated in the experiment. Secondly, we compare in detail the measured conversion efficiency with and without graphene. The power of converted idler without graphene is observed to be ~5.8 dB lower than the one with graphene. We also clarify that the enhanced conversion efficiency mainly comes from the high nonlinearity of graphene. Additionally, the converted idler wavelength can be flexibly tuned by changing the pump wavelength and the conversion efficiency varies slightly. Lastly, for phase conjugated Nyquist 16-QAM wavelength conversion based on degenerate FWM, the observed OSNR penalties are around 1, 1.4 and 1.4 dB at a BER of 1 × 10^−3^ with the converted idler generated at 1547.71, 1546.12 and 1544.52 nm, respectively. For transparent wavelength Nyquist 16-QAM conversion based on non-degenerate FWM, the observed OSNR penalty is around 1.6 dB at a BER of 1 × 10^−3^ with the converted idler generated at 1548.11 nm. It is expected that graphene-assisted nonlinear optical devices might find more interesting optical signal processing applications.

## Methods

**Figure 8:**
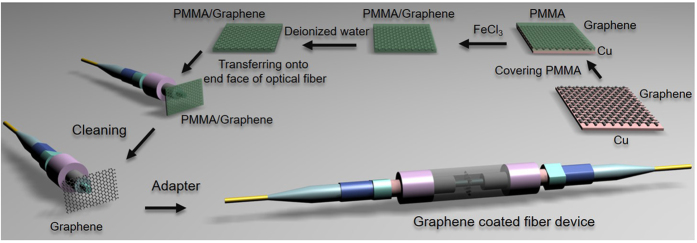
Fabrication process of the graphene-assisted nonlinear optical device.

**Figure 9:**
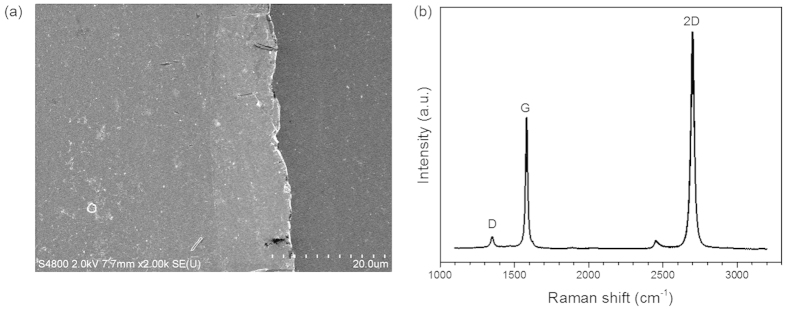
(**a**) SEM image of graphene transferred on silicon-on-insulator (SOI). (**b**) Typical Raman spectrum of single-layer graphene on a SiO_2_/Si substrate (excitation wavelength: 532 nm).

To fabricate the nonlinear optical device based on a single-layer graphene, as shown in Fig. 8, monolayer graphene is first grown on a Cu foil by the CVD method^39^. Poly (methyl methacrylate) (PMMA) film is next spin coated on the surface of the graphene-deposited Cu foil and the Cu foil is etched away with 1 M FeCl_3_ solution. Then, the floating PMMA/graphene sheet is mechanically transferred onto the fiber pigtail cross-section and dried in a cabinet. After drying at room temperature for about 24 hours, the carbon atoms could be self-assembled onto the fiber end-facet thanks to the strong viscosity of graphene. The PMMA layer is finally removed by boiling acetone. By connecting this graphene-on-fiber component with another clean and dry FC/PC fiber connector, the nonlinear optical device is thereby constructed for degenerate/non-degenerate FWM based wavelength conversion applications. Here, the fiber is standard single mode fiber and serves as the brace. The grown graphene sheet is transferred on silicon-on-insulter (SOI) for SEM characterization as show in Fig. 9(a). Selected Raman spectrum is shown in Fig. 9(b). Strong 2D and G bands are observed, accompanied by a weak D band, at 2698, 1582, and 1351 cm^−1^, respectively. The measured I_2D_ /I_G_ ratio of 1.65 confirms the formation of monolayer graphene^40^. The low D to G peak intensity ratios ~0.08 indicates that the graphene formed on a SiO_2_/Si substrate is almost defect-free^41^.

## Additional Information

**How to cite this article**: Hu, X. *et al.* Phase Conjugated and Transparent Wavelength Conversions of Nyquist 16-QAM Signals Employing a Single-Layer Graphene Coated Fiber Device. *Sci. Rep.* **6**, 22379; doi: 10.1038/srep22379 (2016).
