# Author Correction: Phase Conjugated and Transparent Wavelength Conversions of Nyquist 16-QAM Signals Employing a Single-Layer Graphene Coated Fiber Device

**DOI:** 10.1038/s41598-021-99221-z

**Published:** 2021-09-30

**Authors:** Xiao Hu, Mengqi Zeng, Yun Long, Jun Liu, Yixiao Zhu, Kaiheng Zou, Fan Zhang, Lei Fu, Jian Wang

**Affiliations:** 1 Wuhan National Laboratory for Optoelectronics, School of Optical and Electronic Information, Huazhong University of Science and Technology, Wuhan, 430074 Hubei China; 2 College of Chemistry and Molecular Science, Wuhan University, Wuhan, 430074 Hubei China; 3 State Key Laboratory of Advanced Optical Communication Systems and Networks, Peking University, Beijing, 100871 China

Correction to: *Scientific Reports* 10.1038/srep22379, published online 02 March 2016

This Article describes the extension of our previous work, cited in the Article as Reference 26. The studies are scientifically distinct as they describe different nonlinear optics applications of the same home-made graphene coated fiber device. However, due to the conceptual similarities in the study design and related research questions some of the text has been re-used between these papers.

The following sections are partly overlapping (with the differences reflecting differences between the studies): the Abstract, the first and the last paragraph of Introduction, the first paragraph of Experimental Setup; the first two and the last paragraph of Experimental Results, several sentences in the Discussion and the description of Methods.

